# Loss of the coxsackie and adenovirus receptor contributes to gastric cancer progression

**DOI:** 10.1038/sj.bjc.6604876

**Published:** 2009-01-13

**Authors:** M Anders, M Vieth, C Röcken, M Ebert, M Pross, S Gretschel, P M Schlag, B Wiedenmann, W Kemmner, M Höcker

**Affiliations:** 1 Department of Internal Medicine, Division of Gastroenterology and Hepatology, Charité Medical School, Campus Virchow, Augustenburgerplatz 1, Berlin 13353, Germany; 2 Institute of Pathology, Klinikum Bayreuth, Preuschwitzer Str. 101, Bayreuth 95445, Germany; 3 Institute of Pathology, Charité Medical School, Charitéplatz 1, Berlin 10117, Germany; 4 Department of Internal Medicine II, Technical University of Munich, Ismaninger Str. 22, Munich 81675, Germany; 5 Department of Surgery, DRK Kliniken Berlin Köpenick, Salvador-Allende-Str. 2–8, Berlin 12559, Germany; 6 Department of Surgery and Surgical Oncology, Robert Rössle Clinic Charité, Campus Buch, Lindenberger Weg 80, Berlin 13125, Germany; 7 Max Delbrück Center of Molecular Medicine, Robert-Rössle-Straße 10, Berlin 13125, Germany; 8 Center of Anatomy and Integrative Neuroanatomy, Charité Medical School, Campus Mitte, Charitéplatz 1, Berlin 10117, Germany

**Keywords:** coxsackie adenovirus receptor, gastric cancer, prognosis, migration, invasion

## Abstract

Loss of the coxsackie and adenovirus receptor (CAR) has previously been observed in gastric cancer. The role of CAR in gastric cancer pathobiology, however, is unclear. We therefore analysed CAR in 196 R_0_-resected gastric adenocarcinomas and non-cancerous gastric mucosa samples using immunohistochemistry and immunofluorescence. Coxsackie and adenovirus receptor was found at the surface and foveolar epithelium of all non-neoplastic gastric mucosa samples (*n*=175), whereas only 56% of gastric cancer specimens showed CAR positivity (*P*<0.0001). Loss of CAR correlated significantly with decreased differentiation, increased infiltrative depths, presence of distant metastases, and was also associated with reduced carcinoma-specific survival. To clarify whether CAR impacts the tumorbiologic properties of gastric cancer, we subsequently determined the role of CAR in proliferation, migration, and invasion of gastric cancer cell lines by application of specific CAR siRNA or ectopic expression of a human full-length CAR cDNA. These experiments showed that RNAi-mediated CAR knock down resulted in increased proliferation, migration, and invasion of gastric cancer cell lines, whereas enforced ectopic CAR expression led to opposite effects. We conclude that the association of reduced presence of CAR in more severe disease states, together with our findings in gastric cancer cell lines, suggests that CAR functionally contributes to gastric cancer pathogenesis, showing features of a tumour suppressor.

Gastric adenocarcinoma represents the second-leading cause of cancer-related death worldwide (Parkin *et al*, 2005). The clinical outcome of gastric cancer is critically determined by the local tumour growth as well as the presence of local and distant metastases (Maehara *et al*, 2002; Hohenberger and Gretschel, 2003). For both, invasion and metastatic spread, an impaired adhesion of cancer cells is considered a crucial prerequisite. Although previously, in particular, the adherens junction protein E-cadherin (Christofori and Semb, 1999) has been studied, investigation of tight junctions (TJs) in gastric cancer has become of interest in recent years. Hereby, a decreased presence of TJ proteins has been described for claudins 4, 18, and 23, ZO-1 as well as occludin, in part correlating with poor cancer differentiation (Kimura *et al*, 1997; Katoh, 2005; Lee *et al*, 2005; Sanada *et al*, 2006). An increased presence of TJ proteins when compared with normal gastric mucosa has been described for claudins 1, 3, 4, 5, and 7, particularly in intestinal-type adenocarcinomas (Johnson *et al*, 2005; Resnick *et al*, 2005; Hewitt *et al*, 2006; Soini *et al*, 2006; Wu *et al*, 2006). These observations point to a complex deregulation of TJ proteins in gastric carcinogenesis, being suggestive of a cancer inhibitory role of downregulated TJ proteins. In contrast, upregulation of TJ proteins may account for tumour-promoting functions as suggested previously for claudin 1 in colon cancer (Dhawan *et al*, 2005). A more detailed understanding of the functional impact of TJs in gastric cancer, however, is still missing.

The coxsackie adenovirus receptor (CAR), a transmembrane glycoprotein, had initially been characterised as viral attachment site on the surface of epithelial cells (Bergelson *et al*, 1997). Later on it was identified as a component of the TJ complex, an interacting partner for a number of other TJ proteins, and a regulator of TJ formation (Cohen *et al*, 2001; Sollerbrant *et al*, 2003; Coyne *et al*, 2004; Excoffon *et al*, 2004; Mirza *et al*, 2005; Raschperger *et al*, 2006). On the basis of *in vitro* assays, it has been speculated that loss of CAR weakens intercellular adhesion, increases proliferation, and promotes migration as well as invasion of cancer cells (Okegawa *et al*, 2000, 2001; Bruning and Runnebaum, 2003, 2004; Huang *et al*, 2005; Wang *et al*, 2005). These findings led to the hypothesis of a tumour suppressive role for CAR in human cancers. In line with this hypothesis, reduced presence of CAR was found in advanced cancers in part associated with loss of tumour differentiation, increased infiltration, and a poor prognosis (Rauen *et al*, 2002; Sachs *et al*, 2002; Matsumoto *et al*, 2005; Korn *et al*, 2006; Buscarini *et al*, 2007; Okegawa *et al*, 2007). In gastric adenocarcinomas, an immunohistochemical analysis of cancer tissues from 30 patients and 11 non-cancerous controls showed reduced CAR staining intensities in gastric cancer (Heideman *et al*, 2001).

Intrigued by this observation, we hypothesised that CAR is involved in gastric cancer biology. To test our hypothesis, we determined the presence of CAR in a large series of gastric cancer patients and correlated these data with various clinicopathological patient characteristics. Second, we conducted cell culture experiments to evaluate, whether CAR influences the proliferative, migratory, and invasive capabilities of gastric cancer cells.

## Materials and methods

### Study population and tissues

All tissues were obtained from patients (*n*=196) who had undergone curative gastrectomy (R_0_ resection) between 1995 and 2005. Two of the patients received neoadjuvant chemotherapy and exclusion of these individuals from survival analysis did not influence the results. Written informed consent for experimental biomarker analysis was obtained from all patients before analysis. Patients age ranged from 25 to 87 years (mean 64.2±11.7 years). Out of these patients, 54 patients were lost during follow-up, 1 died of unknown cause, and 3 patients died of reasons not related to gastric cancer (mean follow-up time 25.3±22.5 months). Staging and diagnosis of gastric adenocarcinomas was assessed according to the WHO classification (Hamilton and Aaltonen, 2000) and the TNM classification set out by the International Union against cancer (Wittekind *et al*, 2003). Representative areas of each tissue specimen were chosen for the construction of tissue microarrays. Samples of non-neoplastic mucosa were obtained from regions furthest away from the tumour. In brief, a minimum of six tissue cylinders of 0.6 mm diameter from each tumour-bearing donor block and 12 tissue cylinders (six from antrum and six from corpus mucosa) from corresponding non-cancerous mucosa, constructing recipient blocks of tissue microarrays, each with a mean of 141 tissue cylinders from 8–14 patients were punched. An overall mean of 6.1 spots (s.d. 2.7) for each carcinoma from different tumour areas were eligible for analyses. Four *μ*m sections from the tissue microarrays were mounted on Superfrost-plus slides for the subsequent immunofluorescence and immunohistochemical analysis.

### Immunofluorescence and Immunohistochemistry

Immunofluorescence and immunohistochemical staining were carried out as described previously (Anders *et al*, 2003b). In brief, tissue sections were deparaffinised, rehydrated, and submitted to antigen retrieval by microwave treatment. Anti-CAR (H-300: sc-15405, Santa Cruz Biotechnology Inc., Santa Cruz, CA, USA; 1 : 50) and anti-ZO-1 (Zymed, S. San Francisco, CA, USA; 1 : 300) antibodies served as primary antibodies. For immunofluorescence staining, a FITC-conjugated anti-rabbit antibody or a Cy-3-conjugated anti-mouse antibody were used as secondary antibodies (both from Molecular Probes, Eugene, OR, USA). Multicolour fluorescence microscopy was carried out using a Zeiss Axiophot microscope (Carl Zeiss AG, Jena, Germany). For immunohistochemistry, a biotinylated goat anti-rabbit immunoglobulin (Vector Laboratories, Burlingame, CA, USA; 1 : 400) served as secondary antibody, followed by treatment with streptavidin-biotinylated horseradish peroxidase complex (Vectastain Elite ABC kit, Vector Laboratories). Using diaminobenzidine tetrahydrochloride (Sigma-Aldrich, Munich, Germany), sections were developed and counterstained with haematoxylin. Staining results for CAR were evaluated by estimating the percentage of epithelial cells showing specific immunoreactivity by an expert pathologist (MV), who was blinded for the clinical data. The CAR status was classified as: negative (no immunoreactivity), weak (0–5% positive cells), moderate (5–50% positive cells), or strong (>50% positive cells). Only samples showing moderate or strong immunoreactivity were considered positive (Juttner *et al*, 2006).

### Cell culture and generation of stably transfected cell lines

Gastric cancer cell lines AGS, KATO III, MKN28, and MKN45 were cultured as previously described (Juttner *et al*, 2006). Chinese hamster ovary cells stably transfected with human full-length CAR cDNA (CHO-CAR; a kind gift of Dr J Bergelson, Division of Infectious Diseases, Children's Hospital of Philadelphia, PA, USA) and parental CHO cells were cultured in Ham's F12 medium containing 10% fetal calf serum (FCS). Gastric cancer cell line AGS, showing high CAR expression, were chosen for CAR downregulation. Hereby, a CAR-specific siRNA: CCAAGUACCAAGUGAAGACdTdT or a control siRNA: CACAAAAGUAUCGCGCAAGdTdT cloned into the ‘pSuper’ vector system (Oligoengine, Seattle, WA, USA) were stably transfected into AGS cells using Effectene (Quiagen, Hilden, Germany). Following selection with Puromycin (Sigma-Aldrich), CAR downregulation in a pooled cell population was tested by western blotting. MKN28 and MKN45 cells, showing low CAR level, were selected for CAR overexpression. These cell lines were transfected with a construct in which the human full-length CAR cDNA is expressed under control of the CMV promoter in a pcDNA3.1 expression vector (‘hCARpcDNA3.1’; a kind gift of Dr J Bergelson) or pcDNA3.1 expression vector alone (Invitrogen, Karlsruhe, Germany) using Effectene (Quiagen). Following neomycin selection (Invitrogen) CAR expression in a pooled cell population was determined by real time RT-PCR assay and western blotting.

### Quantitative mRNA determination

Total RNA was isolated using TRIZOL (Invitrogen) and reversely transcribed with Oligo-dT primers and SuperScript II (Invitrogen). cDNA generated from 50 ng of total RNA was used for real-time quantification using gene-specific primers (Anders *et al*, 2003b) and the Brilliant QPCR kit (Stratagene, Amsterdam, The Netherlands) on a Stratagene MX3000P cycler. Quantification was performed by the comparative Δ*C*_T_ method normalising *C*_T_ values to *β*-actin. cDNA derived from CHO and CHO-CAR cells were used as negative and positive controls, respectively.

### Western blotting

Protein lysates were obtained as previously described (Anders *et al*, 2003a). Subsequently, equal amounts of protein lysates were loaded on reducing Laemmli gels, immunoblotted with specific antibodies against CAR (H-300: sc-15405, Santa Cruz Biotechnology), or *β*-actin (Sigma-Aldrich), and detected using the ECL system (Amersham Pharmacia, Piscataway, NJ, USA). Protein lysates of CHO and CHO-CAR cells were used as negative and positive controls, respectively.

### Assessment of gastric cancer cell proliferation

Cells were seeded onto six-well plates (*n*=3 × 10^5^ cells per well) in DMEM containing 10% FCS. After 48 h, cells were detached using trypsin and counted using a haematocytometer counting chamber (VWR International, Darmstadt, Germany). All experiments were performed in triplicate and repeated at least twice. Statistical calculations for relationships between CAR status and cell numbers were carried out using Fisher's exact probability test (GraphPad Prism software, version 4.00; GraphPad Software Inc., San Diego, CA, USA).

### Gastric cancer cell migration

Assessment of directed gastric cancer cell migration was performed using an ‘AP48 48 Well Micro Chemotaxis Chamber’ (Neuro Probe, Gaithersburg, MD, USA). Hereby, 50 000 cells per well in 50 *μ*l FCS-free media were seeded onto the upper part of the chamber, whereas its lower compartment was filled with media containing 10% FCS or serum-free media as a control. After 24 h at 37°C, cells migrated through the filter were fixed with 100% methanol and stained using Diff-Quick reagent (Fisher Scientific, Schwerte, Germany). Non-migrated cells at the upper side of the filter were swiped off. All experiments were performed in triplicate and repeated at least twice.

### Gastric cancer cell invasion

Cells were seeded onto the top of ‘BioCoat Matrigel Invasion Chambers’ (BD Biosciences, Bedford, MA, USA) containing 8 *μ*m pore size PET membranes covered with matrigel matrix. Medium containing 10% FCS was added to the bottom well of the chambers as a chemoattractant, whereas serum-free medium was used as a control. Following 24 h at 37°C and 5% CO_2,_ cells that had invaded the matrigel-coated membrane, located at the lower membrane surface, were fixed and stained by crystal violet containing 10% ethanol. Results were documented at a magnification of × 5. Experiments were performed in triplicate and repeated at least twice.

### Statistical analysis

Statistical calculations were performed using GraphPad Prism software (version 4.00; GraphPad Software Inc.). Relationships between CAR immunopositivity and clinicopathological features were evaluated using Fisher's exact probability test. Survival was determined from the date of surgery to the time of event (recurrence or death) using the Kaplan–Meier method. Statistical significance of differences in cumulative survival curves was evaluated using log-rank tests. Additional parameters besides CAR showing a *P*-value <0.05 in the univariate study were included in multivariate survival analyses using the Cox proportional hazard method (SPSS Software, Chicago, IL, USA).

## Results

### Distribution and presence of CAR in non-transformed and malignant gastric tissues

**Figure 1:**
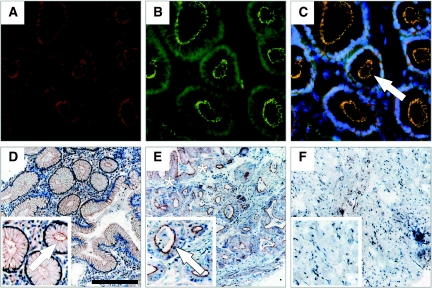
Coxsackie and adenovirus receptor (CAR) presence and distribution in non-transformed gastric mucosa and in gastric adenocarcinomas. Co-immunofluorescence staining for CAR (red **A**, **C**) and ZO-1 (green **B**, **C**) using specific antibodies visualised by phase contrast microscopy: CAR is exclusively detected in epithelial cells, co-localising with ZO-1 at the apical cell surface (arrow in **C**) (magnification: × 400). Typical results for CAR staining determined by immunohistochemistry are shown in non-transformed gastric mucosa, showing CAR localisation at the plasma membrane (arrow in **D**), well differentiated gastric adenocarcinoma, showing apical plasma membrane immunoreactivity (arrow) as well as segmental cytoplasmic staining (asterisks) (**E**), and lack of CAR immunoreactivity in undifferentiated gastric carcinoma (**F**) (magnification: × 100, Bar: 200 *μ*m).

In all samples of non-cancerous gastric mucosa, homogenous CAR immunoreactivity was observed by immunohistochemistry. Coxsackie and adenovirus receptor was restricted to epithelial cells and not found in endothelial, lymphoid, or stromal cells. On a subcellular level, CAR was preferentially localised at the plasma membrane, showing a ‘honeycomb’ appearance typical of junctional staining. Additional immunofluorescence staining for CAR and ZO-1 showed a co-localisation of both proteins at these sites. In contrast, only 56% of gastric adenocarcinoma samples showed CAR positivity (*P*<0.0001). In CAR positive cancers, CAR immunoreactivity was limited to tumour epithelium. On a subcellular level, cytoplasmic immunoreactivity was noted in some areas of the tumour in addition to apical plasma membrane staining (Figure 1).

### CAR presence and clinicopathological parameters

**Figure 2:**
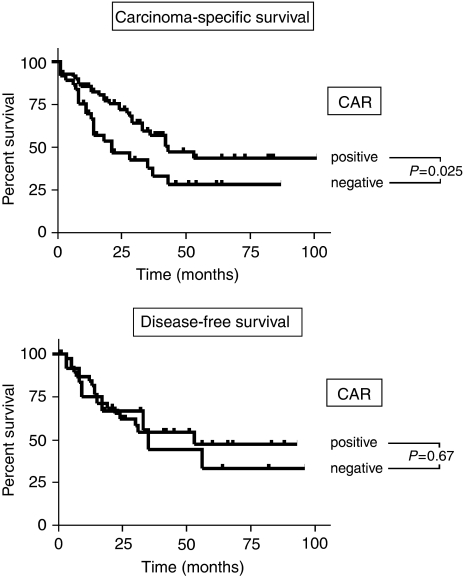
Coxsackie and adenovirus receptor (CAR) presence and clinical outcome of gastric cancer patients. The relationship between CAR and survival of gastric cancer patients was assessed using the Kaplan–Meier method. The upper panel shows results for carcinoma-specific survival, whereas the lower panel represents data for disease-free survival. Statistical evaluation was performed using the log-rank test.

To assess whether loss of CAR correlates with clinicopathological features, we analysed the results for CAR immunoreactivity using the Fisher's exact probability test. Loss of CAR in gastric cancer correlated significantly with decreased differentiation (*P*=0.0238), increased infiltrative depths (*P*=0.0349), and presence of distant metastases (*P*=0.0016). There was no correlation between CAR and tumour types according to Lauren's classification and local tumour spread (Table 1). To evaluate a potential correlation of CAR presence with the survival of gastric cancer patients, the Kaplan–Meyer algorithm was applied. These analyses showed that loss of CAR was associated with shortened carcinoma-specific survival (*P*=0.025). Furthermore, a nonsignificant trend towards shorter disease-free survival (*P*=0.67) in CAR negative cases was observed (Figure 2). Moreover, presence of local and distant metastasis showed a significant association with shortened carcinoma-specific survival, whereas other clinicpathological parameters failed to gain a significant correlation in this analysis (Table 2). The inclusion of patients who died of non-gastric cancer-related causes had no substantial effect on these results (data not shown). Subsequent multivariate analysis showed that CAR does not qualify as independent prognostic factor for carcinoma-specific survival in our cohort. In contrast, presence of lymph node and distant metastases were identified as independent predictors of a poor clinical outcome in this analysis (Table 3).

### CAR expression in gastric cancer cell lines

**Figure 3:**
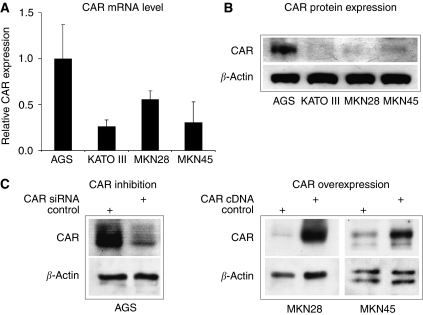
Expression of CAR in gastric cancer cell lines. Real time RT-PCR determination of CAR mRNA levels in gastric cancer cell lines was performed as outlined in the Materials and Methods section. Data represent relative CAR mRNA expression from a series of three independent experiments with CAR mRNA levels in AGS cells set to ‘1’ (**A**). Protein expression levels of CAR and *β*-actin were analysed by western blotting using specific antibodies (**B**). Coxsackie and adenovirus receptor inhibition by stable transfection of CAR-specific siRNA diminished CAR protein expression in AGS cells (left panel), whereas ectopic CAR expression using full-length human CAR cDNA markedly increased CAR protein levels in MKN28 and MKN45 cells (right panel) (**C**).

To evaluate the influence of CAR on gastric cancer biology, we first determined CAR mRNA expression in a panel of four permanent gastric carcinoma cell lines by a real time RT-PCR assay. By normalising *C*_T_ values for CAR expression relative to *β*-actin levels, highest CAR expression was found in AGS cells. In comparison, MKN28 cells showed about two-fold less CAR mRNA expression, whereas in KATO III and MKN45 cells the lowest CAR mRNA values were found (Figure 3A). In contrast, determination of CAR protein expression by western blotting showed a robust signal in AGS cells only. In MKN45 cells a weak signal was detected, whereas no expression was found in MKN28 and KATOIII, potentially due to limitations in sensitivity of the assay (Figure 3B). On the basis of these findings, we chose AGS cells for CAR downregulation, and MKN28 as well as MKN45 cells for CAR over expression in the subsequently performed *in vitro* assays. Western blotting confirmed reduced CAR protein expression in AGS cells upon stable transfection of CAR-specific siRNA, whereas ectopic expression of hCARcDNA in MKN28 and MKN45 cells resulted in a marked increase of CAR protein levels (Figure 3C).

### Impact of CAR on proliferation, migration, and invasion of gastric carcinoma cells

**Figure 4:**
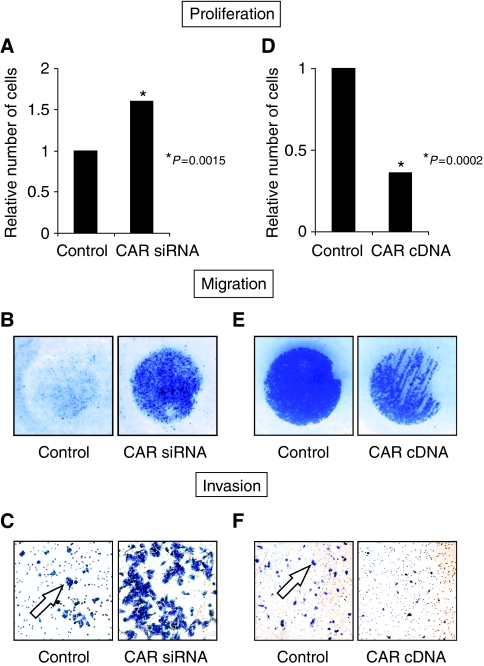
Impact of CAR on gastric cancer cell proliferation, migration, and invasion. The influence of CAR downregulation on proliferation (**A**), migration (**B**), and invasion (**C**) was assessed in AGS cells upon stable transfection of a CAR-specific siRNA compared with the respective ‘vector-only’ control cell line. The impact of CAR upregulation on proliferation (**D**), migration (**E**), and invasion (**F**) was determined in MKN45 (proliferation) and MKN28 cells (migration, invasion) upon stable transfection of a human full-length CAR expression vector ‘hCARpcDNA3.1’. All data represent typical results from a series of three independent experiments. Panels **B** and **E** show characteristic individual wells of a 48 Well Micro Chemotaxis Chamber, as described in the Materials and Methods section. Arrows in panels **C** and **F** indicate clusters of invaded cells. Statistical evaluation was performed by Fisher's exact probability test.

The potential influence of CAR on gastric tumour biology was investigated in a series of *in vitro* experiments. Coxsackie and adenovirus receptor inhibition in AGS cells (AGS^CAR−negative^) yielded significantly higher cell numbers upon 48 h of cultivation in proliferation assays compared with the respective controls (AGS^Vector−control^) (Figure 4A). To minimise the chance of gaining misleading results due to cell death, these cells were counted following staining with Trypan blue dye (Sigma-Aldrich) without finding considerable differences between AGS^CAR−negative^ and AGS^Vector−control^ cells (data not shown). Using an *in vitro* migration assay, AGS^CAR−negative^ cells were found to show markedly increased migratory properties in comparison with AGS^Vector−control^ cells (Figure 4B). To test whether these cells migrate in a FCS-directed manner, FCS-free medium controls were included for each cell line. Hereby, no migration of either AGS^CAR−negative^ or AGS^Vector−control^ cells was noted (data not shown). The evaluation of cancer cell invasion showed a marked increase of AGS^CAR−negative^ *vs* AGS^Vector−control^ cells into matrigel (Figure 4C). In contrast, CAR overexpression in MKN45 cells (MKN45^CAR−positive^) reduced cell proliferation significantly compared with vector-only transfected MKN45 cells (MKN45^Vector−control^) (Figure 4D). The investigation of MKN28^CAR−positive^ *vs* MKN28^Vector−control^ cells did not show significantly different cell numbers. When evaluating these cells in a migration assay, MKN28^CAR−positive^ yielded approximately 50% less migrated cells compared with MKN28^Vector−control^ cells (Figure 4E). Furthermore, a ∼75% reduced invasion of MKN28^CAR−positive^ cells was found compared with MKN28^Vector−control^ cells (Figure 4F). When testing MKN45^CAR−positive^ and MKN45^Vector−control^ cells in these assays, no cell migration or invasion was noted (data not shown).

## Discussion

Here we report for the first time that loss of CAR in gastric adenocarcinomas correlates with reduced tumour differentiation, tumour growth, distant metastases, and reduced survival. In line with these clinical findings, our *in vitro* data show that CAR influences proliferation, migration, and invasion of gastric carcinoma cells.

Our observations show that loss of CAR is not a uniform feature of gastric cancers but correlates with tumour differentiation. So far, claudin 4 has been the only TJ protein shown to be lost in correlation with poor gastric cancer differentiation (Lee *et al*, 2005). Our findings are in line with a limited number of reports showing a correlation between loss of CAR in cancers of the bladder, oesophagus, liver, and pancreas, as well as colon cancers metastatic to the liver (Sachs *et al*, 2002; Matsumoto *et al*, 2005; Korn *et al*, 2006). Whether loss of CAR is a consequence of cancer dedifferentiation, or if CAR itself is involved in the maintenance of epithelial differentiation, however, remains unclear.

Furthermore, we did note a significant correlation between loss of CAR and tumour growth in accordance with observations in bladder cancers made by Sachs *et al* (2002), who found a significant reduction of CAR protein in invasive *vs* superficial tumour specimens, and Okegawa *et al* (2001), who detected significantly lower CAR mRNA levels in stage T3/T4 compared with T1 bladder tumours. Moreover, this study represents, to our best knowledge, the first description of a correlation between loss of CAR and haematogenous spread in human cancer. Considering the role of CAR as cell adhesion molecule, these data support the concept of a disrupted intercellular adhesion as prerequisite for metastasis as described for E-cadherin in gastric cancer (Yonemura *et al*, 1995, 2000). However, our data did not show a correlation between CAR presence and local lymph node spread, as previously observed in bladder cancer (Matsumoto *et al*, 2005). In contrast to these findings and the results of this study, Martin *et al* (2005) suggested that increased CAR levels are associated with the occurrence of breast cancer metastases. Unfortunately, no discrimination between local and distant metastasis is given. Moreover, these data did not reach statistical significance and therefore, need further confirmation. As these authors determined CAR mRNA expression, diverging results maybe also explained by the usage of different techniques.

Our study shows a significant correlation of CAR negativity with reduced carcinoma-specific survival and a trend towards a shorter disease-free survival. These data are in accordance with the pathophysiological concept of an association between a disturbed intercellular adhesion and a poor prognosis in gastric cancer (Yasui *et al*, 2005). Particularly, the loss of E-cadherin has been linked to an unfavourable clinical outcome in gastric cancer patients (Yonemura *et al*, 1995, 2000; Guilford, 1999). However, apart from E-cadherin, only few studies investigated the correlation of TJ proteins with gastric cancer prognosis: reduced expression of claudin 3 has been associated with a poorer prognosis in intestinal-type tumours, yet failing to gain statistical significance in multivariate analysis (Soini *et al*, 2006). Furthermore, gastric cancer patients showing downregulation of claudin 18 had a significantly worse survival, compared with patients with robust expression of this protein (Sanada *et al*, 2006). In contrast, strong expression of claudin 4 significantly correlated with a decreased survival in gastric cancers (Resnick *et al*, 2005). Interestingly, CAR upregulation was correlated with a poor clinical outcome as well: On the basis of CAR mRNA expression, a significant correlation between increased CAR levels and a poor overall survival in breast cancer patients was found (Martin *et al*, 2005). However, as discussed above, these authors measured mRNA expression. This may account for the differences compared with this study and findings in bladder cancer, where loss of CAR expression correlated with decreased cancer-specific survival, but not disease progression, in an univariate analysis (Matsumoto *et al*, 2005).

Given the significant correlation between loss of CAR and advanced disease, as well as reduced survival of gastric cancer patients, it may be speculated that the CAR facilitates an invasion and migration suppressive role in gastric cancer. To clarify whether CAR functionally influences the migratory, and invasive capabilities of gastric cancer cells, we carried out a series of *in vitro* invasion and migration assays upon either inhibition or upregulation of CAR. Hereby, we found that specific CAR silencing increases invasion and migration of gastric cancer cells, whereas ectopic CAR expression resulted in the opposite effect. These *in vitro* findings in conjunction with our observations made in cancer tissue specimens suggest that CAR influences gastric cancer cell migration and invasion, hereby contributing to tumour infiltration and dissemination. Again, our data are in line with the limited number of observations made in other tumour entities: Following retrovirally mediated expression of full-length CAR cDNA in a glioma cell line, cultured in a three-dimensional spheroid model, Huang *et al* observed a reduced cancer cell invasion (Huang *et al*, 2005). An inhibitory effect of CAR on cancer cell migration has been seen upon CAR upregulation in ovarian and cervical cancer cell lines (Bruning and Runnebaum, 2004). The increased migration and invasion of cancer cells upon loss of CAR is currently believed to be a consequence of an impaired intercellular adhesion, as has been shown in ovarian and bladder cancer cell lines (Okegawa *et al*, 2001; Bruning and Runnebaum, 2004; Wang *et al*, 2005). Moreover, it has been suggested that CAR may be involved in processes during reorganisation of the cytoskeleton and thereby impact cell migration and invasion. Hereby, binding of CAR to actin, as been shown previously, may pose a central phenomenon (Huang *et al*, 2007). However, a more detailed understanding of mechanisms underlying functions of CAR in cancer cell motility in general, and gastric cancer cell migration and invasion in particular is currently still missing.

In conclusion, our findings suggest that CAR facilitates tumour suppressor functions in gastric adenocarcinomas. Hereby, our findings add to the current understanding of TJs in gastric cancer, as they represent, to our best knowledge, the first report correlating the presence of a TJ protein with functional characteristics of gastric cancer cells.

## Figures and Tables

**Table 1:** Correlation of CAR immunopositivity with clinicopathological parameters

**Clinicopathological parameters**	**No. of cases**	**CAR positive**	***P*-value**
			**<0.0001**
‘Normal’ mucosa	175	175 (100%)	
Gastric cancer	196	109 (55.6%)	
			
*Cancer type*			0.8634
Intestinal	117	59 (50.4%)	
Diffus	46	24 (52.1%)	
			
*Tumour differentiation*			**0.0238**
G1/G2	69	46 (66.7%)	
G3/G4	127	62 (48.8%)	
			
*Tumour infiltration*			**0.0349**
T1/T2	127	78 (61.4%)	
T3/T4	69	31 (44.9%)	
			
*Lymph node metastasis*			0.6383
N0	57	33 (57.9%)	
N1-3	137	74 (54.0%)	
			
*Distant metastasis*			**0.0016**
Absent	54	40 (74.0%)	
Present	15	4 (26.7%)	

NOTE: statistically significant correlations are shown in bold face. Results were calculated by Fisher's exact test. Divergent numbers of tissue samples assessable for calculations were due to limited accessibility of clinical information, for example, presence of distant metastasis.

**Table 2:** Correlation of CAR immunopositivity and clinicopathological parameters with patients survival

**Clinicopathological parameters**	**Hazard ratio**	**95% CI of ratio**	***P*-value**
*CAR*	1.842	1.090–3.727	**0.0254**
Positive			
Negative			
			
*Cancer type*	1.426	0.6110–3.811	0.3655
Intestinal			
Diffus			
			
*Tumour differentiation*	1.25	0.6883–2.279	0.5432
G1/G2			
G3/G4			
			
*Tumour infiltration*	1.336	0.5772–3.071	0.502
T1/T2			
T3/T4			
			
*Lymph node metastasis*	3.587	1.453–4.808	**0.0015**
N0			
N1-3			
			
*Distant metastasis*	7.129	29.04–1201	**<0.0001**
Absent			
Present			

NOTE: statistically significant correlations are shown in bold face. Survival was determined from the date of surgery to the time of event (death) using the Kaplan–Meier method. Non-cancer-related deaths were excluded from analyses. Statistical significance of differences in cumulative survival curves was evaluated using log-rank tests.

**Table 3:** Multivariate analysis of correlation of CAR immunopositivity and clinicopathological parameters with patients survival

**Clinicopathological parameters**	**Hazard ratio**	**95% CI of ratio**	***P*-value**
CAR	1.310	0,547–3.135	0.545
Lymph node metastasis	2.999	1.077–8.354	**0.036**
Distant metastasis	2.295	1,092–4,824	**0.028**

NOTE: statistically significant correlations are shown in bold face. Parameters showing a *P*-value <0.05 in the univariate analysis were included in the multivariate survival calculation using the Cox proportional hazard method.
